# Decoding the invisible forces of social connections

**DOI:** 10.3389/fnint.2012.00051

**Published:** 2012-07-25

**Authors:** Stephanie Cacioppo, John T. Cacioppo

**Affiliations:** ^1^Department of Psychology, University of GenevaGeneva, Switzerland; ^2^Department of Psychology, University of ChicagoChicago, IL, USA

**Keywords:** social neuroscience, loneliness, bonding, mimicry, synchrony, embodied cognition, interdependence, social isolation

## Abstract

By its 20th anniversary, social neuroscience has witnessed an incredible rise in the number of studies demonstrating the effects of perceived social isolation (e.g., loneliness, ostracism), and inversely, the beneficial effects of social bonding (e.g., love, desire, attachment) on social perception, cognition, and behavior and on mental and physical health. The current review underscores the importance of two factors in this literature: (1) where an individual falls along the continuum of isolation/bonding from feelings of rejection and neglect to feelings of strong, stable, trusted social bonds, and (2) whether gauging an individual's general feeling of social isolation/bonding or the specific feeling of isolation/bonding toward the person with whom the individual is interacting. Evidence shows that these factors are related to brain and cognition, including embodied social cognition—a system integrating past self-related actions from which simulation mechanisms can be used to access other people's minds and anticipate their actions. The neurophysiological mechanisms underlying sensorimotor mapping between interacting individuals offers an empirical opportunity to investigate the interpersonal forces that operate on individuals at a distance. This multilevel integrative approach provides a valuable tool for investigating the brain networks responsible for understanding acute and chronic social disorders.

Social species form organizations that extend beyond the individual. The goal of social neuroscience is to investigate the biological mechanisms that underlie these social structures, processes, and behavior and the influences between social and neural structures and processes (Cacioppo and Berntson, 1992; Cacioppo et al., 2000). The forces operating between individuals to create these superorganismal structures form connections that vary in strength and valence. Whether comparing different individuals at a given point in the lifespan or the same individuals across the lifespan, these social forces vary along a continuum of isolation/bonding from feelings of rejection and neglect to feelings of strong, stable social bonds.

Like the forces between chemical elements, the forces operating between individuals are difficult to observe directly but become visible through their effects on individuals. In the present article, we review some of the visible signs that one can use to identify where individuals fall along the continuum of perceived social isolation/bonding. The traditional way of determining where a person falls along the continuum of perceived social isolation to perceived social bonding is through the use of psychometrically validated questionnaires, such as the UCLA loneliness scale (Russell, 1996). One can also decode social bonds at a distance, for instance, by looking at a person's body language, but doing so involves a multitude of processes that are subject to various other influences. For this reason, validated questionnaires remain the most common and effective way of identifying a person's position on this isolation/bonding continuum. In this review, we focus on the effects on brain and cognition, including embodied cognitive operations such as sensorimotor perception, imitation/mimicry, and interpersonal synchrony. Embodiment here refers to the notion that thoughts, feelings, and behaviors are grounded in sensory experiences and bodily states (for reviews see Semin and Smith, 2002; Niedenthal et al., 2005; Barsalou, 2008; Schubert and Semin, 2009; Meier et al., 2012). We begin by reviewing the effects of perceived social bonding/isolation on health.

## Social isolation/bonding and health

**Figure 1:**
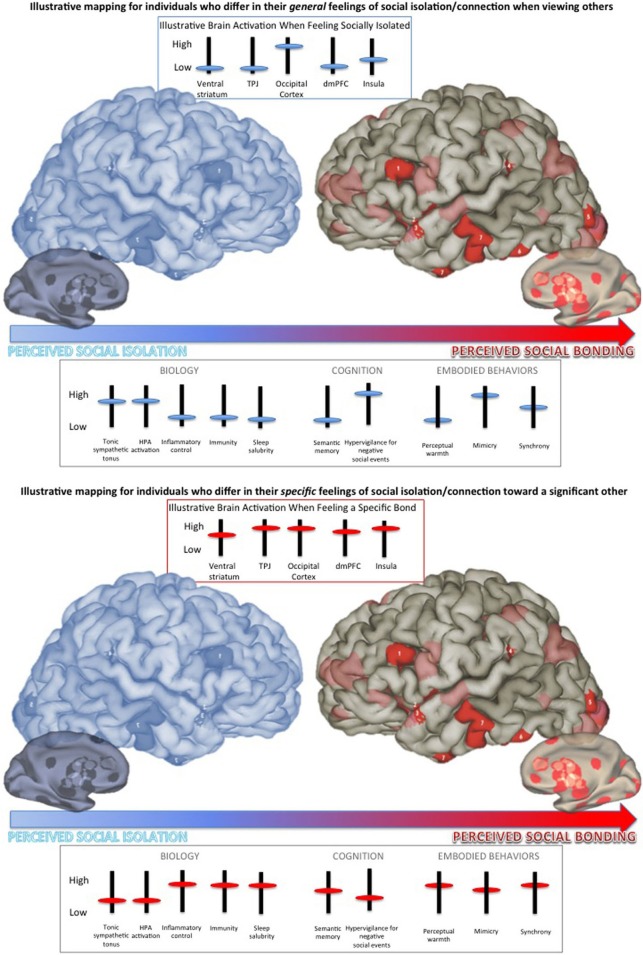
**Schematic representation of the continuum of social isolation/bonding from feelings of social isolation or neglect (in blue) to feelings of strong, salubrious social bonds (in red).** Brain and behavioral responses differ depending on an individual's general feeling of social isolation/connection and specific feeling of isolation/connection to the person with whom one is interacting. **Top Panel**: Illustrative mapping for individuals who differ in their *general* feelings of social isolation/connection when viewing others (see Cacioppo and Patrick, 2008). **Bottom Panel**: Illustrative mapping for individuals who differ in their *specific* feelings of social isolation/connection toward (e.g., love for) a significant other (see Ortigue et al., 2010; Cacioppo et al., 2012; Brain Graphic by James W. Lewis, West Virginia University published in the Scientific American Mind).

A person's position along the continuum of perceived social isolation/bonding to others is associated with a variety of physical and mental health effects (see Figure 1). Perhaps most striking, people who subjectively feel they are isolated or have few if any strong connections to others (in blue on the spectrum; Figure 1) live shorter lives than those who feel they have strong, dependable, meaningful social bonds (Cacioppo and Patrick, 2008; Cacioppo and Cacioppo, 2012; Luo et al., 2012; Perissinotto et al., 2012). The increased risk of mortality is evident even when objective social isolation and health behaviors are statistically controlled (Luo et al., 2012). Meta-analyses of the odds ratio for increased mortality for perceived social isolation/bonding in humans is 1.45—larger than found for marriage or physical activity, approximately double the odds ratio for increased mortality for obesity, and quadruples the odds ratio for air pollution (Holt-Lunstad et al., 2010).

Perceived isolation in humans, as measured using the UCLA loneliness scale, and the experimental manipulation of social isolation in nonhuman animals have been associated with a number of effects, including increased hypothalamo-pituitary adrenocortical activation, tonic sympathetic tonus, depressive behavior, and prepotent responding, and decreased inflammatory control, viral immunity, and expression of genes regulating glucocorticoid responses (Figure 1, **top panel**; see review by Cacioppo et al., 2011). Both cross-sectional and longitudinal studies have also demonstrated that perceived isolation in humans increases sleep fragmentation and daytime fatigue (Cacioppo et al., 2002, 2011).

Interestingly, animal work suggests that social connection constitute a stimulus that can have direct effects not only on physical and mental health but on brain structures and function. Consider the desert locust (Schistocerca gregaria) as a case in point. The desert locust has an asocial and a social state. The asocial state is the more typical condition, during which period the locust tends to avoid conspecifics. Under specifiable conditions, however, the locusts transform from a solitary to a swarming phase, at which point the brains of these locusts grow approximately 30% larger, presumably to accommodate the additional information processing demands of their now more complicated social environment (Ott and Rogers, 2010). The deprivation of these social connections leads to a return to the asocial phase, along with a consequent reduction in brain volume.

Social processes were once thought to have been incidental to human learning and cognition, but the social complexities and demands of primate species are now thought to have contributed to the evolution of the neocortex and various aspects of human cognition (Dunbar and Shultz, 2007; Dunbar, 2011, 2012). In line with this reasoning, cross-species comparisons have revealed that the evolution of large and metabolically expensive brains is more closely associated with social than ecological complexity (Dunbar and Shultz, 2007). Moreover, although human toddlers and chimpanzees have similar cognitive skills for engaging and interacting in the physical world, toddlers show more sophisticated cognitive skills than chimpanzees for engaging the social world (Hermann et al., 2007).

For any member of a social species, it is dangerous to be on the social perimeter (Cacioppo and Patrick, 2008). Social species can vary in terms of the position along a continuum of social isolation (e.g., neglect, exclusion) to social connection or bonding. In nonhuman animals, where an individual falls along this continuum is typically manipulated experimentally by housing the animal in isolation or with conspecifics for an extended period of time. Given the complex social ties that characterize human existence, the irrepressibly meaning-making nature of humans, and the ethical constraints against experimentally isolating individuals for an extended period, a large literature has developed showing that *perceived* social isolation in normal samples is a more important predictor of a variety of adverse behavioral, psychological, and health outcomes than is objective social isolation. For instance, where an individual falls along the continuum of perceived social isolation/bonding—whether acute or chronic—may also have important consequences for cognitive abilities. Feeling socially isolated or excluded appears to increase attention to social information, especially negative information. For instance, research shows that people, who feel socially rejected, show an increase in memory for selected social information (Gardner et al., 2000), and are more sensitive to emotional vocal tone and are more accurate on a facial emotion detection task (Pickett et al., 2003) than people who feel accepted by a group. Studies using the social Stroop task have also shown that the interference in the Stroop task produced by negative social words is a direct function of how socially isolated the participants feel, whether the feelings of social isolation were experimentally manipulated (acute) or dispositional (chronic; Cacioppo and Hawkley, 2009; see, also, Powers and Heatherton, 2012; Tsukiura, 2012).

Importantly, prospective longitudinal studies of older adults also show that perceived isolation is a risk factor for general cognitive decline (e.g., Tilvis et al., 2004) and Alzheimer Disease (Wilson et al., 2007). Illustrative of the latter is a large prospective study conducted by Wilson et al. (2007) in 823 older adults free of dementia at enrollment. They found that the more the participants felt socially isolated, the poorer their later cognitive performance in semantic memory, perceptual speed, and visuo-spatial skills (compared to baseline as assessed by an extensive battery of cognitive measures). Furthermore, Cox proportional hazards models that controlled for age, sex, and education indicated that perceived social isolation significantly increased the risk of clinical Alzheimer Disease: 76 individuals developed dementia during the 65 month study period. This association was unchanged when objective social isolation, depressive symptomatology, or other demographic and health-related factors served as covariates.

## Social isolation/bonding and brain mechanisms

From a neuro-functional viewpoint, recent evidence from both human and nonhuman animal studies investigating the biochemistry and brain activity associated with social isolation/bonding point to specific patterns of activation elicited by social stimuli. Cacioppo et al. (2009) identified a specific brain signature associated with perceived isolation in a brain imaging study in which participants performed a categorical judgment task. In the scanner, participants viewed pictures chosen from the International Affective Picture System (IAPS) that varied in their emotional (i.e., negative/unpleasant, positive/pleasant) and social (i.e., nonsocial, social) content, and participants specified whether each picture was pleasant, neutral, or unpleasant. Results showed that the closer participants were to the social isolation anchor of the continuum, the greater the activation of the ventral striatum to pleasant nonsocial, in contrast to social pictures, whereas the closer participants were to the social bonding anchor, the greater the activation of the ventral striatum to the pleasant social, in contrast to nonsocial, pictures.

Individuals who fell near the socially isolated end of the continuum showed greater activity in the dorsal mPFC to pleasant nonsocial, relative to social, stimuli, whereas individuals who fell near the socially bonded end of the continuum showed the greatest activity in this region to pleasant social, compared to nonsocial, stimuli. Prior functional neuroimaging work on thinking about the characteristics of people (e.g., Jenkins et al., 2008) and deciding to be altruistic toward another person (Waytz et al., 2012) has reliably shown the dorsal mPFC to be involved. Together, these data fit the notion that the more individuals feel socially isolated from others, the greater the emphasis on self-preservation and maintaining a safe psychological distance from others.

For unpleasant pictures, the closer participants were to the social isolation anchor of the continuum, the greater the activation of the visual cortex to *social*, in contrast to nonsocial pictures, whereas the closer participants were to the social bonding anchor, the less the difference in the activation of the visual cortex to the social and nonsocial pictures. These neuroimaging data parallel the behavioral findings from the social Stroop task. It is dangerous on the social periphery. Humans who feel socially isolated and nonhuman animals who are experimentally isolated increase behaviors that promote predator evasion and self-preservation. Interestingly in this context, the closer participants were to the social isolation anchor of the continuum, the less the difference in the activation of the temporo-parietal junction to social, in contrast to nonsocial pictures, whereas the closer participants were to the social bonding anchor, the greater the activation of the temporo-parietal junction to the social and nonsocial pictures—consistent with the notion that the former are more likely to focus on self-preservation and, therefore, reflect less on the perspective of others in a negative social context.

Powers et al. (2012) extended these results by reinforcing the role of the dMPFC in social isolation/bonding during the processing of social and non-social stimuli. Powers et al. manipulated social exclusion in 32 female undergraduates, who then viewed social and non-social pictures selected from the IAPS, and categorized them as indoor or outdoor scene. Their results revealed that the dmPFC was significantly modulated by social exclusion. Consistent with Cacioppo et al. (2009), socially excluded participants showed no differences in activation of the dmPFC for social and nonsocial scenes, whereas socially included participants showed greater dmPFC activity to social than non-social scenes.

Thus far, we have dealt with regional brain activation in individuals who vary in their feeling of social isolation/bonding in response to pictures of *unfamiliar* people in positive or negative circumstances (Figure 1, **top panel**). A related literature has emerged investigating the regional brain activation in individuals who vary in their feeling of social isolation/bonding in response to pictures of a specific significant other (Figure 1, **bottom panel**). This work suggests that the feeling of love (disdain) for and closeness to (distance from) a significant other elicits both common and unique neural processes.

Brain mapping of individuals who feel social bonding with a significant other activates the subcortical brain areas that are associated with euphoria, reward, and motivation as well as the cortical brain areas that are involved in social cognition and self-representation (such as anterior cingulate cortex, middle frontal gyrus, superior temporal gyrus, precentral gyrus, temporo-parietal junction, and occipo-temporal cortices; Ortigue et al., 2010; Cacioppo et al., 2012). The deactivation of subcortical dopaminergic-rich areas during experiences of social isolation/bonding is in line with psychological studies defining social connections as a rewarding, positive, and motivating experience. Interestingly, the co-activations of these subcortical emotion-related areas with cortical areas that mediate more complex cognitive functions (e.g., social knowledge, mentalizing, body image, mental associations, and self-awareness and understanding others) reinforces the top-down neuro-functional model of interpersonal relationships, which suggest that associative cortical regions may be priming the emotion-related areas and visual cortex to be more sensitive to certain kinds of information—in essence, instructing the eyes on what kind of person is perceived as socially positive or negative, and telling the emotional centers what to feel. From these results, one may consider social isolation/bonding on a spectrum that calls for a hypo- to hyper-activation of the same network for social bonding.

Interestingly, a growing body of neuroimaging studies suggests several overlapping areas (e.g., prefrontal areas, insula) between the network sustaining social isolation/bonding, and that sustaining embodied cognitive behaviors. As a distinct knowledge domain, embodied cognition recruits a bilateral network of cortical brain regions including this inferior fronto-parietal network (i.e., inferior parietal lobule, inferior frontal gyrus) as well as the bilateral posterior superior temporal sulcus, dorsal premotor cortex, and ventral premotor cortex (Grafton, 2009). Within this bilateral network, embodied cognition acts as a special knowledge system with dedicated encoding and retrieval processes, which play a role in the interaction between what we do and what we perceive. Along these lines, it makes sense that the way individuals perceive others and their connections with others may also modulate the way they perceive their actions, imitate them and/or synchronize with others.

Within the brain network sustaining embodied behaviors, the discovery of the inferior fronto-parietal mirror neuron system (MNS), which includes a type of neurons (i.e., mirror neurons) that are activated both by the execution and the observation of object-related actions, may play a role in mimicry, synchrony, and embodied behaviors more generally (see Semin and Cacioppo, 2009 for review). Neurophysiological and functional neuroimaging studies suggest the existence of a motor resonance mechanism in the premotor and the posterior parietal cortices that is activated during motor imitation (Jackson et al., 2006) and when participants observe goal-directed actions executed by another individual (e.g., Grafton et al., 1996; Jackson et al., 2006). These data have generally been interpreted as evidence for the direct-matching hypothesis, which states that we understand actions by mapping the visual representation of the observed action onto a sensorimotor representation (Rizzolatti and Craighero, 2004; Rizzolatti and Sinigaglia, 2008; Semin and Cacioppo, 2009). If the MNS is involved in the embodied signs of social isolation/bonding, as has been postulated, then the activation of this system should not be seen simply as a response to an observed action but should be powerfully modulated by the nature of the social connection between the actor and observer. How this brain network is modulated as a function of where the individuals fall along the continuum of perceived social isolation/bonding is an open question at this point. We turn next to behavioral research on this question.

## Social isolation/bonding and sensorimotor perception

Recent research on embodied cognition has shown that feelings of social warmth or coldness can be induced by experiences of physical warmth or coldness, and vice versa. This is consistent with a growing body of research on embodied cognition as well as work underscoring the centrality of interpersonal warmth (vs. coldness) in person perception (Asch, 1946; Kelley, 1950; Cacioppo and Gardner, 1999; Bargh and Shalev, 2012). One explanation for the power of the warm-cold dimension in person perception is that somatosensorial experiences (such as temperature perception) constitute an “embodied ground” for social proximity and abstract and psychological concepts and metaphors (such as interpersonal warmth; Asch, 1958; see also Semin and Smith, 2008; Bargh and Shalev, 2012 for reviews). For instance, people often describe their feelings as “warm” when they are thinking about a trustworthy and loving individual and “cold” when they are thinking about a detached, distant individual (Asch, 1946; Fiske et al., 2007; IJzerman and Semin, 2010).

Interestingly, where people fall along the social isolation/bonding continuum has been shown to be related to their estimates of the room temperature. For instance, Ijzerman and Semin (2009) found that participants seated in a warm room reported feeling interpersonally closer to the experimenter compared to participants seated in a colder room. Together these studies show that experiences of physical warmth produce concomitant feelings of social warmth. Reciprocally, IJzerman and Semin (2010) showed that physically (or verbally) induced experiences of closer social proximity/warmth produced changes in the perception of room, and led to higher estimates of room temperature. On the other end on the continuum of perceived social isolation/bonding, Zhong and Leonardelli (2008) demonstrated that individuals who felt socially isolated estimated room temperature to be lower than those who felt socially bonded, and they also showed greater desire for warm food (hot soup) and drinks (hot coffee), but not for the two control foods (apples and crackers) and the control drink (icy soda). Bargh and Shalev (2012) hypothesized that individuals who feel socially isolated might tend to self-regulate their feelings of social warmth through applications of physical warmth. Consistent with this reasoning, Bargh and Shalev found significant positive associations between perceived social isolation and both the frequency of bathing and the typical duration of a bath or shower, as well as a trend for individuals who felt socially isolated to prefer warmer water temperature. In sum, there is growing evidence that the association between physical warmth/coldness and social warmth/coldness share a common representation or code (Meyer-Lindenberg, 2008).

## Social isolation/bonding and mimicry/synchrony

The idea of embodiment and behavior matching in social settings is not new. Scholars have long observed that people tend to mirror the emotional and motor expressions of others (Smith, 1759/1976; James, 1890/1950; Hatfield et al., 1994). For instance, it has been shown that couples who have been married for a long period of time tend to resemble each other in their expressions and actions more than random couples of the same age, and married couples resemble each other even more than they did when they were first married (Zajonc et al., 1987; Mondillon et al., 2007). Studies of motor and emotional contagion also illustrate how people automatically mimic others (e.g., contagious yawning and laughter, body inclination, for a review, see Hatfield et al., 1994). The nature of the connection linking these individuals has been found to matter, however (Lakin and Chartrand, 2005; Lakin et al., 2008). Lakin et al. (2008), for instance, found that people who felt excluded by an in-group mimicked a confederate who was an in-group member more than a confederate who was an out-group member (Lakin et al., 2008).

The ability of individuals to automatically mimic others has been assumed to facilitate the transmission of known behaviors from one individual to another (and so from one generation to the next), and also to be involved in the discovery and incorporation of innovative behaviors into a group's behavioral repertoire. In this sense, imitation is thought to facilitate social learning, cohesion and tradition (the transmission of known behaviors among individuals; Hatfield et al., 1994). Accordingly, it has been suggested that imitation of close others might serve the adaptive function of increasing affiliation, liking, and rapport between people (see Hatfield et al., 1994; Lakin and Chartrand, 2005).

Interpersonal *mimicry* refers to the similarity in form of the actions between individuals, whereas interpersonal *synchrony* refers to the coordination of movement that occurs between individuals, featuring both similarity in form and the temporal alignment of the actions. As illustrated by the Social Cognition model (from Semin and Cacioppo, 2009), synchronization is “time-locked to the observed stimulus.” Like mimicry, interpersonal synchrony increases the social connection felt between individuals. For instance, synchrony has been shown to facilitate relationship formation (Vacharkulksemsuk and Fredrickson, 2012), to improve group cohesion (McNeil, 1995), to foster cooperation (Wiltermuth and Heath, 2009), and to breed compassion (Valdesolo and DeSteno, 2011), emotional support satisfaction (Jones and Wirtz, 2007), elevated pain thresholds (Cohen et al., 2010) and affiliation (Hove and Risen, 2009). There is some evidence that the affiliative effects are not dependent on an individual's awareness of the interpersonal synchrony (e.g., see review by Hatfield et al., 1994).

According to emotional contagion theory (Hatfield et al., 1992, 1994), people spontaneously mimic facial and bodily expressions, especially with whom they feel a close social connection to the person (Hatfield et al., 1994; Mondillon et al., 2007). Consistent with this notion, recent research shows that people who are “psychologically experiencing self-other overlap as a result of self-disclosure” are more likely to synchronize their body movements (Vacharkulksemsuk and Fredrickson, 2012). Social motivation plays an important modulating role. For instance, Lakin and Chartrand (2005) showed that individuals who feel socially isolated/excluded, and who therefore are motivated to create new connections with others, mimic strangers more than people who do not feel socially excluded. Subsequent research indicates that people who feel socially isolated not only display greater mimicry with a stranger, but they show an advantage in decoding nonverbal cues (e.g., fake smile vs. real smile; Bernstein et al., 2008) and, specifically, cues that may indicate rejection (Pickett and Gardner, 2005).

In sum, embodied mechanisms are not a pre-requisite to act, connect or understand others, but the extant literature suggests that embodied behaviors offer new ways to investigate social perception, cognition, and behavior (e.g., Semin and Smith, 2002; Semin and Cacioppo, 2009; Schubert and Semin, 2009; Meier et al., 2012). Aron and Aron's (1986) self-expansion model, which posits that others toward whom one feels a close social bond can be incorporated into the representation of one's self, and the relational model of communal sharing and cognitive interdependence (see Fiske, 2004; Smith, 2007; IJzerman and Semin, 2010), which posits that self-representations that incorporate aspects of others also foster interdependent behavior, are consistent with the notion that social bonds are grounded in people's actions. Recent advances in the neurosciences make it possible to investigate whether an individual's position along the continuum of social isolation/bonding modulates shared sensorimotor representations and visible embodied behaviors.

### Conflict of interest statement

The authors declare that the research was conducted in the absence of any commercial or financial relationships that could be construed as a potential conflict of interest.
